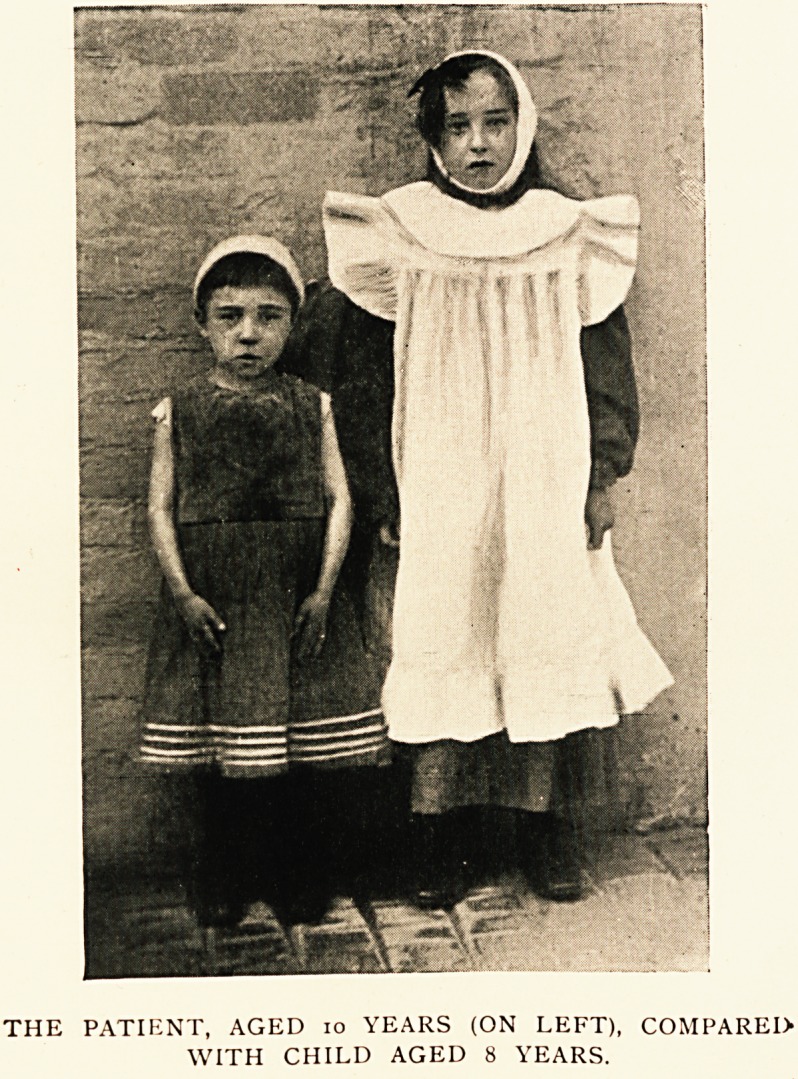# Notes on a Case of Infantilism
2Paper read before the Society for the Study of Disease in Children.


**Published:** 1904-09

**Authors:** E. Cecil Williams

**Affiliations:** Physician in charge of Out-Patients, Bristol Royal Hospital for Sick Children and Women


### NOTES ON A CASE OF INFANTILISM.[2]

BY

E. CECIL WILLIAMS, B.A., M.B. Cantab.,

*Physician in charge of Out-Patients, Bristol Royal Hospital for Sick Children and Women.*

THE child is aged 10 years. Her parents state that since four years of age she has not grown to any appreciable degree. The father and mother are both well grown and intelligent people.

[2] Paper read before the Society for the Study of Disease in Children.

I have seen two sisters, who are of normal size. The child was brought under my care at the Children's Hospital because of her small size and lack of development. Her weight is 26 lbs. (average weight, about 4 st. 6 lbs.); height, 36 inches (average, 4 ft. 3 ins.); circumference of head, 18¾ ins. (average, 21½ ins.); length, 12 ins.; breadth, 10½ ins. The primary teeth are decaying. The fontanelles are closed. The child is naturally silent and slow, awkward to manage at times, and is rather dirty in her habits. She can read her letters and count. She answers simple questions fairly intelligently. There is nothing to account for this condition; no heart lesion; no rickets; no congenital syphilis; no symptoms of pancreatic insufficiency. She digests cod liver oil well. For a few weeks I gave her some thyroid extract; her mother thought she was brighter when taking it, but it had to be discontinued, as she lost 4½ lbs. in weight. Mr. Hastings Gilford,[1] in his paper on "Ateleiosis," a disease characterised by conspicuous delay of growth and development, divides his cases into three groups:

1. Appearing before birth.
2. During infancy.
3. In the later stages of development.

The facial expression and general appearance of these cases must vary with the age of onset of the disease. The word infantilism has been used by many writers in cases where it is only a symptom, as in some cases of congenital syphilis or of congenital heart disease. Mr. Gilford, however, refers to a condition which he calls "normal infantilism," and says, "We recognise that growth may vary greatly, and that it may sometimes be excessive without being morbid; just in the same way as there may be normal giants, so there may be normal dwarfs; and in some of these dwarfs there is not only delay or arrest of growth, but also of development. The chief point of difference between normal infantilism and ateleiosis is that development is arrested in a much lesser degree in infantilism than ateleiosis, and that these cases eventually may arrive at maturity. At this stage I do not think it possible to say

[1] *Med.-Chir. Tr.*, 1902, lxxxv. 305.

THE PATIENT, AGED 10 YEARS (ON LEFT), COMPARED WITH CHILD AGED 8 YEARS.

definitely whether this child should be regarded as a case of ateleiosis or of normal infantilism. I am disposed to favour the latter. If classed as an ateleiotic, she should certainly be placed in group 3, as the disease came on after the first dentition. Some ateleiotics live to a good old age. With regard to the pathology of the condition, I do not intend to offer any suggestions. Whether the disease is a primary disease of the skeleton, or whether it is dependent on some abnormality of the sexual organs, pituitary body, pancreas, or thyroid gland, is fully treated in Mr. Gilford's paper which I have already quoted.

## Figures and Tables

**Figure f1:**